# Dual roles of *in situ* generated HSP70 in antigen delivery and immunoregulation

**DOI:** 10.3389/fimmu.2025.1638948

**Published:** 2025-10-02

**Authors:** Xinliang Kang, Zhuofan Li, Jayachandra Reddy Nakkala, Yibo Li, Labone Akter, Yiwen Zhao, Xinyuan Chen

**Affiliations:** Biomedical and Pharmaceutical Sciences, College of Pharmacy, University of Rhode Island, Kingston, RI, United States

**Keywords:** HSP70, antigen delivery, immunoregulation, inflammation, radiofrequency adjuvant, immunomodulation

## Abstract

**Introduction:**

Extracellular release of inducible HSP70 spurred interests to explore its potential interactions with innate immune systems. Both pro- and anti-inflammatory roles have been reported though the immunostimulatory roles were largely disputed due to the likely use of contaminated HSP70. The anti-inflammatory roles inspired the exploration of HSP70 to treat autoimmune diseases by suppressing pathological inflammatory responses. Besides immunomodulation, HSP70 has been explored as tumor vaccine carriers to elicit cytotoxic T lymphocyte responses due to its ability to deliver bound peptides to MHC I presentation pathway. With increasing understanding of the potential use of ex vivo prepared HSP70 in vaccination and therapy, the functions and potential applications of *in situ* induced HSP70 in antigen delivery and immunomodulation remain largely unexplored.

**Methods:**

This study utilizes physical radiofrequency adjuvant (RFA) to induce HSP70 synthesis accompanied with mild inflammation followed by intradermal injection of vaccine antigens into RFA-treated skin in murine models to explore its potential roles in antigen delivery and immunomodulation.

**Results:**

We found *in situ* induced HSP70 could bind intradermally injected model antigen ovalbumin and contribute to enhanced antigen uptake in skin and draining lymph nodes. HSP70 failed to induce dendritic cell maturation and rather suppressed RFA-induced TLR4/IRAK/NFκB activation and IL-6 expression.

**Discussion:**

These results indicate dual roles of *in situ* induced HSP70 in antigen delivery and immunoregulation at physiological conditions. These dual functions highlight opportunities to exploit endogenous HSP70 for both vaccine adjuvantation and immunomodulation.

## Introduction

Heat-shock proteins (HSPs) are molecular chaperones that exist in all living organisms and are essential for cell survival (1). HSPs bind nascent proteins to aid in folding during translation. HSP levels can be significantly increased under stress to aid in the folding of unfolded or misfolded proteins and promote cell survival (1). HSPs are overexpressed in many cancer types and are often associated with poor prognosis (2). HSPs are classified into different families based on molecular weights (e.g., HSP40, HSP70, HSP90) (3). HSPs of different families share little amino acid sequence homology, while HSPs of the same families are highly conserved from prokaryotes to eukaryotes (3). Mammalian HSPs can be found in different cellular compartments, such as cytosol, endoplasmic reticulum, and mitochondria (3).

HSP70 is one of the most extensively explored HSPs (4). Mammalian HSP70 can be divided into constitutively expressed (hereafter HSc70) – always produced or inducible one (HSP70) – only produced under stress (4). HSP70 was able to deliver bound peptides to MHC I presentation pathway and elicit cytotoxic T lymphocyte (CTL) responses (5, 6). It was found that HSP70/peptide complexes purified from tumor but not normal tissues could induce CTL responses and anti-tumor immunity (5, 6). Physical HSP70/peptide association was the key to generate anti-tumor immunity considering HSP70 or peptides alone could not elicit similar responses (5, 6). The ability of HSP70 to aid associated peptides to induce CTL responses and anti-tumor immunity was shared by other HSPs, such as HSP90 and GP96 (7). These exciting findings inspired clinical investigation of HSP-based cancer vaccines against various tumor types, including melanoma, pancreatic cancer, colon cancer, and glioblastoma (8, 9). HSP-based cancer vaccines were well tolerated and showed the most efficacy against early-stage tumors and in high vaccine-dose groups (8, 9). The ability of mammalian HSP70 to aid bound peptides to induce CTL responses and anti-tumor immunity *in vivo* was also shared with Mycobacterium tuberculosis HSP70 (10, 11). Besides potentiation of CTL responses, HSP70 of Mycobacteria, murine, porcine, and human origins has been found to also enhance humoral immune responses against associated vaccine antigens *in vivo* (11–14), indicating the facilitated delivery of associated peptides also to MHC II presentation pathway (15, 16).

Besides antigen delivery, extracellular HSP70 was reported to stimulate DC maturation and activate monocytes, macrophages, and DCs to release cytokines (17–19). A new term ‘chaperokine’ was coined to describe the dual functions of HSP70 to serve as chaperone and cytokine (20). Various receptors, such as CD14, TLR2, TLR4, and CD91, were reported to bind HSP70 and mediate its cytokine function (17, 21, 22). These findings were subsequently disputed by the use of highly purified HSP70, which lacked the ability to induce strong DC maturation or cytokine release (23, 24). Despite the efforts to remove endotoxin, the high affinity of endotoxin to HSP70 and the high potency of endotoxin to stimulate cytokine release might contribute to the cytokine function of HSP70 (23, 24). Other contaminations, such as lipoproteins, might also contribute to the cytokine function of HSP70 (23, 24). Consistently, none of the receptors (CD14, TLR2, TLR4, CD91) on antigen presenting cells (APCs), capable of binding endotoxin or lipoprotein, were able to bind highly purified HSP70 and instead scavenger receptor LOX-1 was found to bind HSP70 with high affinity (25).

Different from its immune-stimulation, evidence emerged in the last 2 decades that mammalian HSP70 possessed immunoregulatory functions and could suppress LPS and TNFα-induced inflammatory responses (26–28). Consistently, Mycobacterium tuberculosis HSP70 showed anti-inflammatory roles in proteoglycan-induced arthritis animal models (29, 30). Mechanism studies found mammalian and Mycobacterium HSP70 could modulate DC function to stimulate the production of IL-10-producing T cells and IL-10 has been critical for HSP70-induced protective roles (29, 31, 32). Regulatory T cells have been also key to a conserved HSP70 peptide (B29)-induced protection in arthritis models (30). These studies inspired the exploration of HSP70 to treat autoimmune diseases and transplantation rejection (33, 34).

HSP70 can be induced under various stress conditions, for example, thermal stress, oxidative stress, environmental toxins (35–37), after radiofrequency tumor ablation (38), or after non-ablative skin rejuvenation (39). Besides the promotion of cell survival under stress conditions, potential roles of *in situ* produced HSP70 in the context of antigen delivery and/or immunoregulation remain largely unexplored. In addition, prior studies largely focused on exploring antigen delivery or immunomodulation and rarely explored both functions at the same time. We recently found non-ablative radiofrequency (RF) treatment of mouse skin could induce significant HSP70 synthesis (40), which played crucial roles in RF adjuvant (RFA) effects to boost intradermal (ID) vaccination against influenza (41). RFA also induced transient local inflammation (40). RFA treatment creates a favorable local environment and enables the simultaneous exploration of the impact of *in situ* induced HSP70 on antigen delivery and immunomodulation in physiological conditions. By intradermally injecting model antigen ovalbumin (OVA) into RFA-treated skin, this study explored whether the *in situ* induced HSP70 could release into extracellular space, bind ID antigens, and facilitate their intracellular delivery. At the same time, this unique model allows the exploration of the potential roles of *in situ* induced HSP70 on local inflammation.

## Materials and methods

### Sex as a biological variable

Our previous studies found sex was not a biological variable for RFA effects (42). This study exclusively used male mice for consistency. We expect the same results in female mice.

### Reagents

Endotoxin-free ovalbumin (OVA) and AddaVax were purchased from Invivogen (San Diego, CA). Fluorescence-conjugated antibodies were purchased from Biolegend (San Diego, CA) or otherwise specified. Adenosine 5′-diphosphate (ADP)-Agarose (A2810-5ML) was obtained from Sigma-Aldrich (St. Louis, MO). Goat polyclonal antibody against mouse/rat MyD88 (AF3109) was purchased from R & D Systems (Minneapolis, MN). Rabbit polyclonal antibody against human/mouse/rat TIRAP (PA5-88657) and Alexa Fluor 647-conjugated OVA (AF647-OVA, O34784) were obtained from Thermo Fisher Scientific (Waltham, MA). FITC-conjugated anti-HSP70 antibody (C92F3A-5, ab61907) and recombinant mouse HSP70 (ab113187) were purchased from Abcam (Cambridge, MA). HSP70 ELISA kit (ADI-EKS-715) was purchased from Enzo Life Sciences (Farmingdale, NY).

### Mice

C57BL/6 mice (6–8 weeks old, male) were purchased from Jackson Laboratory (Bar Harbor, ME). TLR2 knockout (KO) (004650), TLR4 KO (029015) and Myd88 KO mice (009088) were obtained from Jackson Laboratory (Bar Harbor, ME) and self-bred for use in this study. Heterozygous HSP70 KO mice were originally obtained from the Mutant Mouse Resource & Research Centers (MMRRC) at University of Missouri and self-bred to obtain HSP70 KO mice for use in this study. Animals were housed in facilities of University of Rhode Island (URI) and anesthetized by intraperitoneal injection of a mixture of 80 mg/kg Ketamine and 10 mg/kg Xylazine for hair removal, RF treatment, and immunization. In most of the experiments, hair on the lateral back skin was removed by shaving followed by topical application of hair removal lotion (Nair) one day before experiment. Mice were euthanized by CO_2_ inhalation. All animal procedures were approved by the Institutional Animal Care and Use Committee (IACUC) of the University of Rhode Island (AN1516-004) and conducted in accordance with National and Institutional Guidelines and Regulations. Animal experiments were reported in accordance with the ARRIVE guidelines.

### RF treatment

A handheld fractional bipolar RF device (~1 MHz, high-energy setting, Norlanya Technology Co., Hong Kong, China) equipped with 12 × 12 array of microelectrode in 2 × 2 cm^2^ was used to treat mouse skin for 1–2 min without causing visible or histological skin damages as indicated in our previous study (40). Before treatment, a thin layer of ultrasound coupling medium was applied on the skin surface and RF device was firmly pressed to enable treatment tips to have a tight contact with the skin. For sham treatment, ultrasound coupling medium was applied and RF device was firmly pressed except the device was not activated.

### Intracellular HSP70 staining and flow cytometry

Lateral back skin of C57BL/6 mice were exposed to RF or sham treatment. RF or sham-treated skin (12 × 12 mm^2^) was dissected at indicated time points followed by digestion in collagenase D (0.2%) and dispase (0.6 U/mL) for preparation of single-cell suspensions as in our previous report (43). Skin cells were then stained with fixable viability dye eFluor 450 (65-0863-14, Thermo Fisher Scientific) and then fluorescence-conjugated CD11b (clone M1/70), CD11c (clone N418). Cells were then fixed and permeabilized, and further stained with fluorescence-conjugated anti-Hsp70 (C92F3A-5) followed by flow cytometry analysis in BD FACSVerse. Flow cytometry data were analyzed using FlowJo™ software (version 10).

### Skin HSP70 purification and silver staining

Purification of HSP70 from mouse skin referred to a published protocol with slight modification (7). RF or sham-treated mouse skin was homogenized in 1.5 ml hypotonic buffer (10 mM NaHCO_3_, 0.5 mM PMSF, pH 7.1) followed by centrifugation at 12,700 rpm for 10 min. Supernatants were collected and changed to buffer D (20 mM Tris-acetate, 20 mM NaCl, 15 mM β-mercaptoethanol, 3 mM MgCl_2_, 0.5 mM PMSF, pH 7.5) using PD-10 column (GE Healthcare Life Sciences, Marlborough, MA). Samples were then concentrated with Centrifugal Filter Unit (Amicon^®^, 10 kDa cutoff) to 0.5 mL and applied to an ADP-Agarose column equilibrated with buffer D. The column was washed with buffer D containing 0.5 M NaCl and then buffer D. The column was then incubated with 0.5 mL buffer D containing 3 mM ADP at room temperature for 30 min. Finally, column was eluted with 1mL buffer D containing 3 mM ADP. HSP70 (RF) or HSc70 (sham)-rich elutes were resolved on sodium dodecyl sulfate-polyacrylamide gel electrophoresis (SDS-PAGE) followed by silver staining via a commercial kit (24612, Thermo Fisher Scientific).

### Western blotting

HSP70 (RF) or HSc70 (sham)-rich elutes were resolved on SDS-PAGE followed by transferring to polyvinylidene difluoride (PVDF) membrane. After blocking with 5% non-fat milk, PVDF membrane was incubated with mouse anti-HSP70 antibodies (1:1000, MA1-10889, Invitrogen) (no cross-reactivity with HSc70) or rabbit anti-HSc70 polyclonal antibodies (1:500, AB1427, Abcam) (no cross-reactivity with HSP70) at 4˚C overnight. After washing in Tris-buffered saline (TBS) containing 0.05% Tween 20 (TBST), PVDF membrane was incubated with HRP-conjugated anti-mouse (1:5000, NA931, GE Healthcare Life Sciences) or anti-rabbit secondary antibodies (1:1000, 7074P2, Cell Signaling Technology) at room temperature for 1 h. After washing in TBST, PVDF membrane was incubated with Pierce ECL Western Blotting Substrate (32109, Thermo Fisher Scientific). PVDF membrane was imaged under Imager (Thermo Fisher Scientific).

### Immunoprecipitation/immunoblotting

Skin was homogenized in RIPA buffer for exploration of cytoplasmic protein interactions or membrane lysis buffer (50 mM Tris, pH 7.5, 150 mM NaCl, 0.1% NP40, 0.05% CHAPS, 1 mM PMSF, and protease inhibitor) for exploration of protein interactions involving membrane proteins as recommended (44). The same amounts of total proteins were incubated with anti-OVA (ab181688, Abcam), -TIRAP (PA5-88657, Thermo Fisher Scientfic), or -IRAK4 antibodies (MA515883, Thermo Fisher Scientific) at 4 °C for 1 h. Protein A/G agarose was then added and incubated at 4 °C overnight. Supernatants were removed after centrifugation. Protein A/G agarose was then washed with RIPA or membrane lysis buffer 3 times. Sample buffers were added, boiled, and centrifuged. Supernatants were subjected to SDS-PAGE and IB detection of HSP70 (MAB1663, R & D Systems), TLR4 (sc-293072, Santa Cruz), and IRAK1 (Ab238, Abcam).

### *Ex vivo* skin culture

The lateral back skin of C57BL/6 mice was exposed to RF or sham treatment. RF or sham-treated skin (12 × 12 mm^2^) was dissected right after treatment, cut into ~1 mm wide slices, and cultured in 200 μl RPMI 1640 complete medium at 37 °C in a 5% CO_2_ incubator for 18 hr. Culture medium was then harvested, centrifuged, and supernatants were used for measurement of HSP70 levels. Skin tissues were harvested at the same time, homogenized in T-PER buffer, and centrifuged. Supernatants were used for measurement of skin HSP70 levels.

### Extraction of interstitial fluids with laser-based powder reservoir patch

We took advantage of our recently developed reservoir powder patches topically applied onto ablative fractional laser (AFL) treated skin to extract tissue fluids for quantification of extracellular HSP70 levels (45). Briefly, mannitol powder-coated cylindrical reservoir patches (8mm in diameter and 5mm in depth) were prepared by loading an excessive volume of mannitol powder over the blank reservoir patches followed by centrifugation at 20,000 rpm for 90 min. Powder above the surface of the reservoir patches was removed by a scalpel. Approximately 200 mg of mannitol could be coated per reservoir patch. Powder reservoir patches were kept in a desiccator before use. Lateral back skin of mice was exposed to RF or sham treatment. RF and sham-treated skin was then subjected to AFL treatment (5mJ energy, 10% coverage) to generate skin microchannels. Powder reservoir patches were then topically applied and further sealed with Tegaderm film to form an air-tight system. A narrow bandage was used to keep the entire system in position. Reservoir patches were removed 24 h later. At this moment, powder mannitol became a wet mass due to absorption of interstitial fluid through laser-generated skin microchannels. The wet mannitol was transferred to an Eppendorf tube and completely dissolved in 0.5 mL phosphate-buffered saline (PBS). Sample volume was reduced to ~100 µl via Centrifugal Filter Unit (Amicon^®^, 3 kDa cutoff) for measurement of HSP70 levels.

### Quantification of HSP70 levels

HSP70 levels in tissue homogenates or extracted interstitial fluids were quantified by HSP70 High Sensitivity ELISA kit (ADI-EKS-715, Enzo Life Sciences), which specifically detects inducible HSP70. Briefly, diluted samples were added to ELISA plates coated with monoclonal antibody specific for inducible murine HSP70 and incubated at room temperature for 2 h. After washing, polyclonal antibody specific for inducible murine HSP70 was added and incubated at room temperature for 1 h. After washing, HRP-conjugated secondary antibodies were added and incubated at room temperature for 1 h. TMB substrate was added after plate washing and incubated at room temperature for 30 min. Stop solution was lastly added and plates were read at 450nm in a microplate reader (Molecular Device).

### Skin nuclear fraction extraction

Nuclear fractions of RFA, Sham, and LPS-treated skin were extracted with a commercial tissue nuclear extraction kit (Abcam, AB219177). In brief, skin was cut into small pieces followed by homogenization in 1 mL cold Cytoplasm Extraction Buffer supplemented with protease inhibitor and DTT. After thorough homogenization, skin homogenates were transferred to an Eppendorf tube and incubated on ice for 10 min. Skin homogenates were vortexed and centrifuged at 1,000 g for 3 min. Supernatants (the cytoplasmic portion) were carefully and completely removed. Pellet was resuspended in 750 µL Nuclear Extraction Buffer supplemented with protease inhibitor and DTT and incubated on ice for 15 min. Tubes were vortexed every 5 min during the incubation followed by centrifugation at 5,000 g for 3 min. Supernatants were collected for use as soluble nuclear fraction in our studies.

### ELISA antibody titer

Serum antibody titer was measured by enzyme-linked immunosorbent assay (ELISA). In brief, 96-well ELISA plates were coated with 10 µg/ml for OVA at 4˚C overnight. After blocking with 5% non-fat milk, 2-serial dilutions of immune sera were added and incubated at room temperature for 90 min. After washing in PBS supplemented with 0.05% Tween 20 (PBST), HRP-conjugated sheep anti-mouse IgG secondary antibodies (1:5,000, NA931, GE Healthcare Life Sciences) were added and incubated at room temperature for 1 hr. After washing in PBST, TMB substrates were added and reactions were stopped by addition of 3M H_2_SO_4_. Optical absorbance (OD_450/630 nm_) was read in a microplate reader (Molecular Device). Serum antibody titers were defined as the reciprocal dilution factor that resulted in OD_450/630 nm_ that was ~3 times higher than the background value.

### Skin IL-6 cytokine levels

Skin was homogenized in RIPA buffer supplemented with protease inhibitor and PMSF (1 mM). Supernatants were collected after centrifugation. Total protein levels were measured by BCA assay. IL-6 levels were measured by a commercial ELISA kit (431304, BioLegend, San Diego, CA). Briefly, ELISA plates were coated with capture antibodies at 4°C overnight. Plates were washed and then blocked at room temperature for 1 h. Samples were added and incubated at room temperature for 2 h. Plates were washed and detection antibodies were added and incubated at room temperature for 1 h. Plates were washed and avidin-HRP was added and incubated at room temperature for 30 min. After washing, substrate was added and reactions were stopped 20 min later. Plates were read at OD_450/570 nm_.

### BMDC culture

Bone marrow was isolated from femur and tibia of C57BL/6 mice and cultured in the presence of recombinant murine GM-CSF (15 ng/ml) and IL-4 (10 ng/ml) as in our previous report (46). Immature DCs were harvested on day 6 and seeded into at 10^6^ cells/mL in 96-well plates and then incubated with AF647-OVA in the presence of LPS, HSP70-rich elute, HSc70-rich elute or the same volume of medium. Cells were then harvested 20 hr later, stained with fllig;uorescence-conjugated anti-CD11c (N418), CD40(3/23), CD80 (16-10A1), and CD86 antibodies (GL-1) followed by flow cytometry analysis of AF647+ cells in CD11c+ cells and mean fluorescence intensity (MFI) of CD40, CD80, and CD86 in AF647+CD11c+ cells.

### Antigen uptake and DC maturation

WT, HSP70 KO, TLR2 KO, TLR4 KO, and MyD88 KO mice were subjected to RFA or Sham treatment followed by ID injection of 2 µg AF647-OVA into RFA or sham-treated skin. Skin and draining lymph nodes (LNs) were dissected 20 hr later after euthanasia. Single-cell suspensions of the skin were prepared by collagenase D (0.2%) and dispase (0.6 U/mL) digestion. LNs were passed through 40 μm cell strainers to prepare single-cell suspensions. Skin and LN cells were then stained with fluorescence-conjugated anti-CD11c (clone N418), MHC II (clone M5/114.15.2), Langerin (clone 4C7), CD11b (clone M1/70), CD103 (clone 2E7), and CD80 antibodies (clone 16-10A1) followed by flow cytometry analysis.

### Proximity ligation assay

Lateral back skin of C57BL/6 mice was intradermally injected with 5 µg LPS (LPS-EB Ultrapure, tlrl-3pelps, Invivogen) or subjected to RF or sham treatment. Skin was dissected 6 hr later and cryo-sectioned followed by PLA via a commercial kit (Duolink^®^ *In Situ* Red Starter Kit Goat/Rabbit, DUO92105, Sigma-Aldrich). In brief, frozen sections were recovered to room temperature, fixed in 3.7% paraformaldehyde, and permeabilized in PBS supplemented with 10% goat serum and 0.1% Triton-X 100. Sections were then blocked and incubated with goat anti-mouse MyD88 polyclonal antibodies and rabbit anti-mouse TIRAP polyclonal antibodies at 4˚C overnight. After washing, sections were incubated with Probe Minus and Plus from the kit at 37˚C for 1 hr. After washing, sections were subjected to ligation at 37˚C for 30 min and then amplification at 37˚C for 100 min. After final washing, sections were mounted with Duolink^®^ *In Situ* Mounting Media with DAPI mounting medium (DAPI) and cover slipped. Images were taken under a Nikon Ti2-E confocal microscope. The number of positive PLA signals in randomly selected skin area of 0.4 mm^2^ was counted and compared between groups. Only PLA signals in close vicinity to cell nucleus were counted to avoid false signals.

### Statistics

Values were expressed as Mean ± SEM (standard error of mean). Student’s t-test was used to compare differences between groups and one-way ANOVA with Tukey’s Multiple Comparison test was used to compare differences for more than 2 groups except otherwise specified. P value was calculated by PRISM software (GraphPad, San Diego, CA) and considered significant if it was less than 0.05.

## Results

### RFA vigorously induces HSP70 expression in DCs

**Figure 1:**
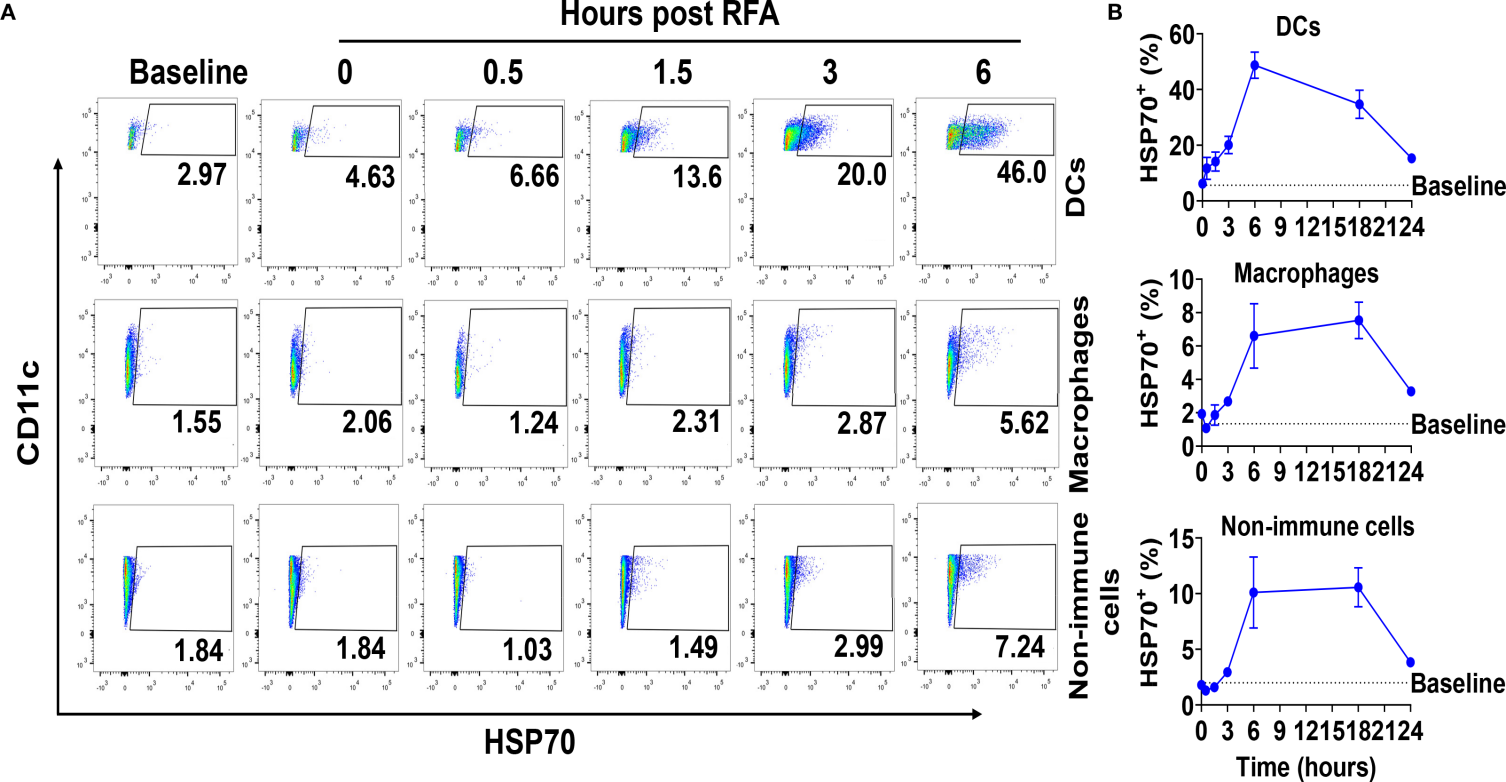
RFA vigorously stimulates HSP70 synthesis in DCs. C57BL/6 mice were subjected to RFA treatment. Single-cell suspensions were prepared at different timepoints followed by staining with fluorescence-conjugated viability dye, anti-CD11c, and anti-F4/80 antibodies. Cells were then permeabilized followed by intracellular staining with fluorescence-conjugated anti-HSP70 antibodies. **(A)** Representative dot plots showing dynamic increase of percentage of HSP70+ cells in DCs, macrophages, and non-immune cells in the first 6 h after RFA treatment or non-treated skin (baseline). **(B)** Percentage of HSP70+ DCs, macrophages, and non-immune cells at different timepoints till 24 h n=4. Data are representative of two independent experiments with similar results.

Our previous studies found that RFA could increase skin HSP70 levels 6 and 24 h after treatment (40). Here, we further explored the temporal and cell type-specific induction of HSP70 following RFA treatment. Skin cells were divided into three major types based on CD11c and F4/80 expression (**Supplementary Figure S1A**): DCs (CD11c^+^ F4/80^-^), macrophages (F4/80^+^), and non-immune cells (CD11c^-^ F4/80^-^). RFA rapidly induced HSP70 expression in DCs while showed a delayed effect to induce HSP70 expression in macrophages and non-immune cells. In support, the percentage of HSP70^+^ DCs rapidly increased as early as 0.5 h after RFA treatment, but the percentage of HSP70^+^ macrophages and non-immune cells showed no significant increase in the first 1.5 h after RFA treatment (**Figures 1A, B**). RFA also induced the most HSP70 expression in DCs. In support, the percentage of HSP70^+^ DCs reached a peak level of 49% at 6 h, while the percentage of HSP70^+^ macrophages and non-immune cells reached a peak level of 6-8% and 10-11% between 6–18 h, respectively (**Figure 1B**).

### RFA-induced HSP70 can be purified and separated from HSc70

**Figure 2:**
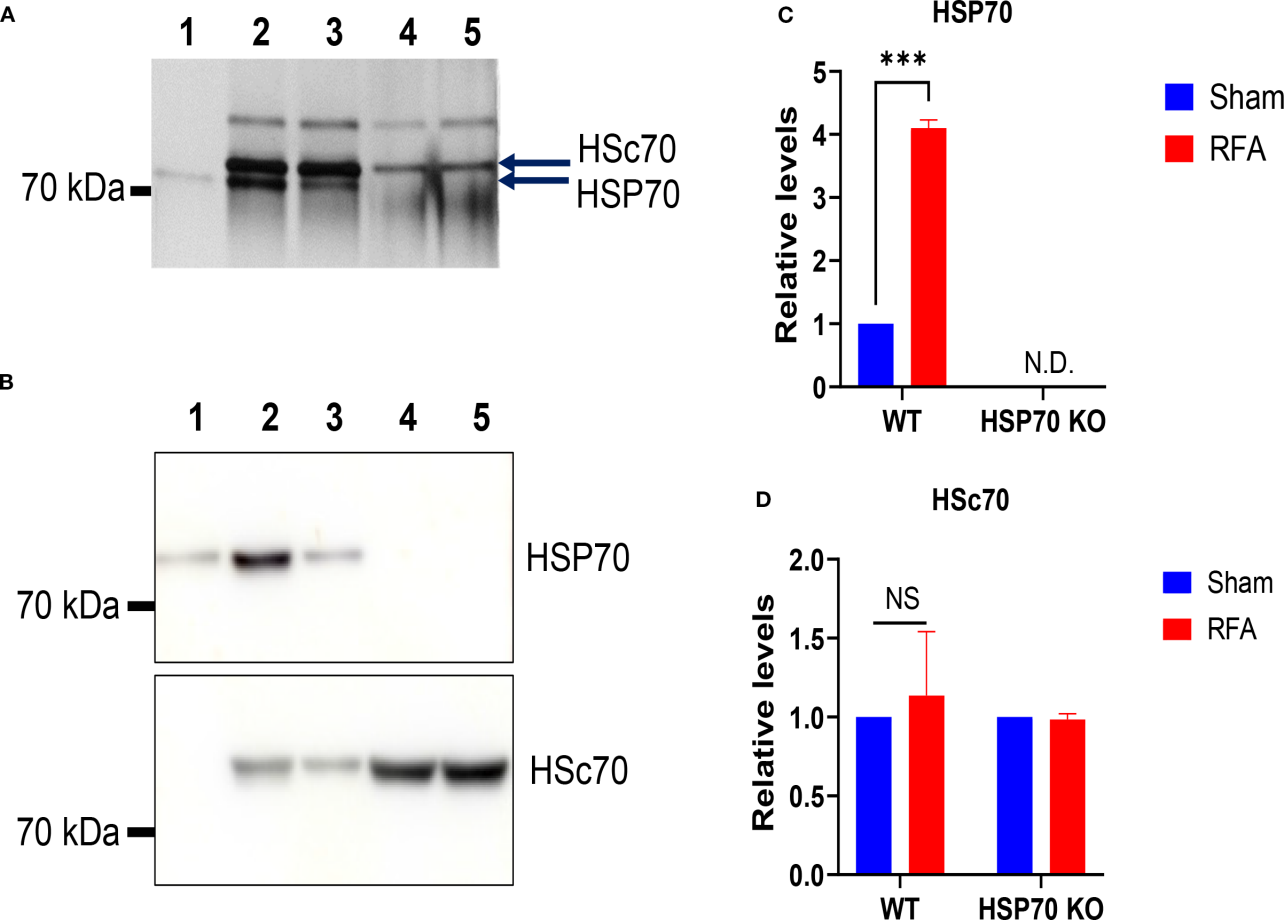
Separation of RFA-induced HSP70 from constitutively expressed HSc70. **(A)** Lateral back skin of WT and HSP70 KO mice were subjected to RFA or Sham treatment. RFA and Sham-treated skin (1×1 cm^2^) was collected 18 h later, homogenized, and purified with ADP-Agarose column. Eluted samples were concentrated and adjusted to the same volume of 100 µl. The same volume (10 µl) of WT samples isolated from 4 pieces of skin, KO samples isolated from one piece of skin, and recombinant murine HSP70 were subjected to SDS-PAGE separation and silver staining. **(B)** WT (10 µl) and KO samples (30 µl), both purified from one piece of skin, and recombinant murine HSP70 were subjected to SDS-PAGE separation and western blotting analysis with anti-HSP70 antibodies (upper) or anti-HSc70 antibodies (lower). The volume of KO samples was increased for readiness detection of HSP70 if there was any. 1. Recombinant mouse HSP70; 2. Purified sample from RFA-treated WT skin; 3. Purified sample from Sham-treated WT skin; 4. Purified sample from RFA-treated HSP70 KO skin; 5. Purified sample from Sham-treated HSP70 KO skin. Full membrane pictures were shown in **Supplementary Figure S2**. **(C, D)** Densitometry analysis of relative HSP70 **(C)** and HSc70 levels **(D)** of **Figure 2B** in WT and HSP70 KO mice using ImageJ. Results were the combination of two independent experiments with similar results. Two-way ANOVA with uncorrected Fisher’s LSD test was used to compare differences between Sham and RFA groups. N.D., not detectable. NS, not significant. ***, p<0.001.

Next, we explored whether RFA-induced HSP70 could be purified by ADP-Agarose column due to the ability of HSP70 to bind ADP through its N-terminal nucleotide-binding domain (NBD) (7). In this study, silver staining was used to detect HSP70 after SDS-PAGE due to its relatively low amount. Also, recombinant murine HSP70 (full-length, inducible) and purified samples from HSP70 KO mice were included to facilitate the identification of inducible HSP70. As shown in **Figure 2A**, two bands with molecular weight slightly higher than 70 kDa were found in purified WT samples, while only one band with the higher molecular weight was found in purified HSP70 KO samples. Additionally, the lower-molecular-weight bands in purified WT samples matched the recombinant murine HSP70. These data hinted the lower-molecular-weight bands were inducible HSP70, while the higher-molecular-weight bands were constitutively expressed HSc70. More intense HSP70 band in RF group of WT mice hinted RF treatment induced its synthesis.

Western blotting was further used to confirm the above findings using anti-HSP70 and anti-HSc70 antibodies with no cross-reactivity to each other. As shown in **Figure 2B**, anti-HSP70 antibodies recognized the lower-molecular-weight bands in purified WT samples and also recombinant murine HSP70, while anti-HSc70 antibodies recognized the higher-molecular-weight bands in both WT and HSP70 KO samples. We also found that RF treatment significantly induced HSP70 expression in WT but not HSP70 KO mice (**Figures 2C, D**). Our results indicated RF-induced HSP70 could be separated from constitutively expressed HSc70 in SDS-PAGE despite their small difference in molecular weights as reported (47).

### Extracellular release of RFA-induced HSP70

**Figure 3:**
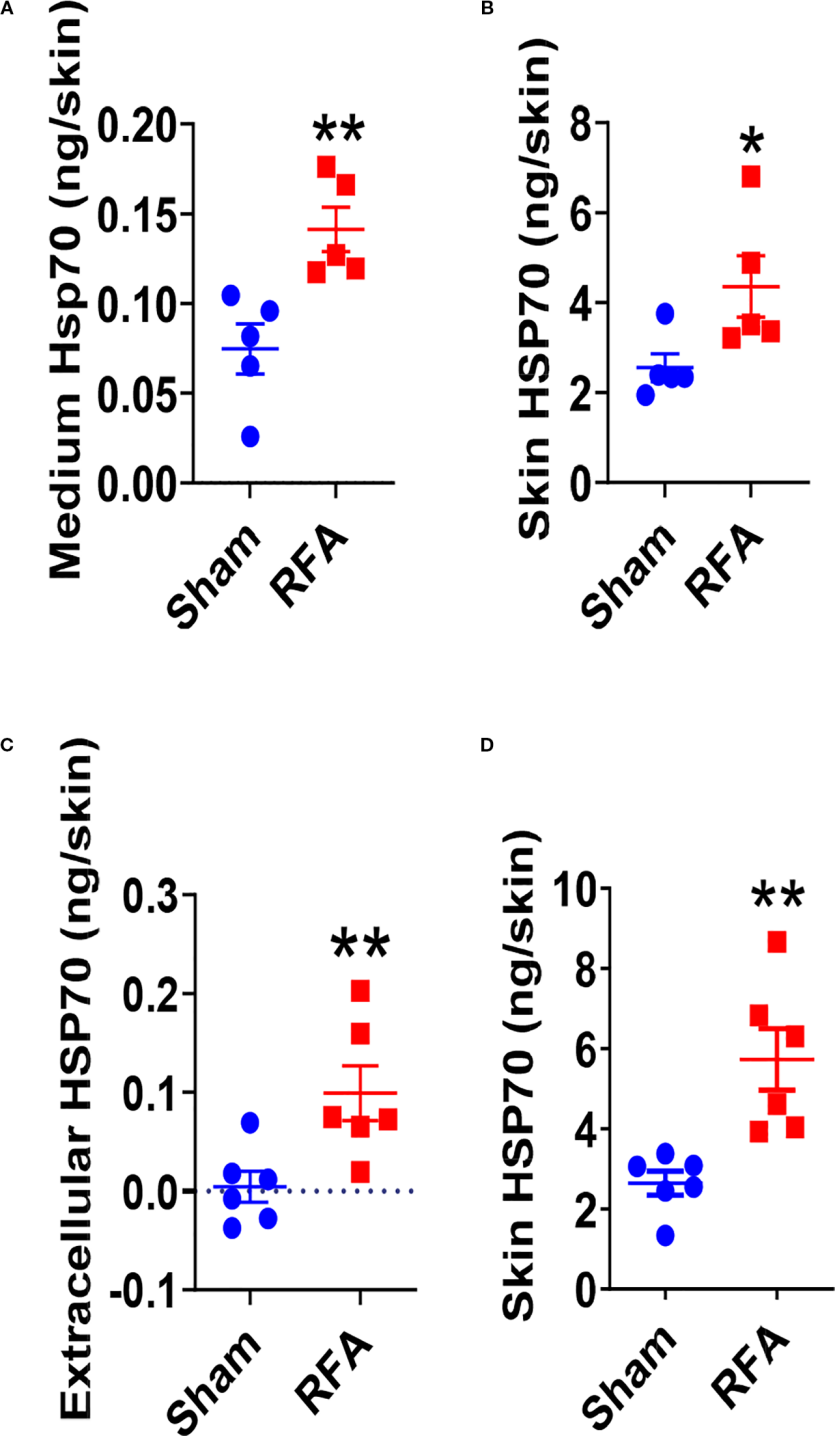
RFA stimulates HSP70 synthesis and extracellular release. **(A, B)** Lateral back skin (1.2×1.2 cm^2^) of C57BL/6 mice was subjected to RFA or Sham treatment. Skin was dissected right after treatment and further cut into 1 mm wide slices for culture in 24-well plates. A small volume (100 µl) of medium was added to just cover the tissue. Culture medium was harvested 18 h later to measure HSP70 levels **(A)**. Skin was also harvested at the same time and homogenized in T-PER buffer for measurement of HSP70 levels **(B)**. **(C, D)** Lateral back skin (1.2×1.2 cm^2^) of C57BL/6 mice was subjected to RFA or Sham treatment. Skin was then subjected to one pulse of AFL treatment at 5mJ energy and 10% coverage. Powder mannitol-coated reservoir patches were then topically applied to extract interstitial fluid via skin microchannels. Powder reservoir patches **(C)** and underneath skin **(D)** were harvested 24 h later for measurement of HSP70 levels. n=5 in **A, B** and n=6 in **(C, D)** Two-tailed student’s t-test was used to compare differences between groups. *, p<0.05; **, p<0.01. Data are representative of two independent experiments with similar results.

HSP70 was reported to export extracellularly via non-canonical pathways to modulate immune system function (48, 49). Next, we explored whether RFA-stimulated HSP70 could be exported into extracellular space. We first conducted *ex vivo* skin culture and measured HSP70 levels in the culture medium. As shown in **Figure 3A**, significantly higher HSP70 levels were detected in the culture medium of RFA-treated skin than that of Sham-treated skin (0.14 vs. 0.075ng). RFA-treated skin also showed significantly higher HSP70 levels than Sham-treated skin (4.4 vs. 2.6 ng) (**Figure 3B**). This result indicated RFA could stimulate HSP70 synthesis with significant release into extracellular space. Interestingly, low-level HSP70 could be detected in Sham-treated skin, in line with our previous report (40).

Next, we used another method recently developed in house to extract interstitial fluids for measurement of extracellular HSP70 levels. This method took advantage of ablative fractional laser (AFL)-generated skin microchannels and topically applied bulk mannitol powder to absorb interstitial fluids followed by dissolution for convenient measurement of secreted HSP70 levels (45). As shown in **Figure 3C**, HSP70 levels in interstitial fluids of RF-treated skin was much higher than that of sham-treated skin (0.1 vs. 0.004 ng). HSP70 levels were also significantly higher in RF-treated than sham-treated skin (5.7 vs. 2.6 ng, **Figure 3D**). These data correlated well with the *ex vivo* skin culture data, supporting RF stimulated HSP70 synthesis with a significant extracellular release. Considering HSP70 may also be exported as a membrane-bound form, we further used cmHSP70 antibodies (kindly provided by Dr. Gabriele Multhoff) (18, 50) to specifically identify membrane bound HSP70 after RFA or Sham treatment. As shown in **Supplementary Figure S3**, a small percentage (<1%) of skin cells showed positive membrane HSP70 binding after RFA treatment, which showed no significant difference from that after sham treatment. This result indicated extracellular HSP70 following RF treatment mainly existed as a soluble but not membrane-bound form. Interestingly, we failed to detect HSP70 in blood stream even after bilateral RF treatment (data not shown), hinting RF mainly induced localized HSP70 release.

### Evidence of *in situ* HSP70/antigen association

**Figure 4:**
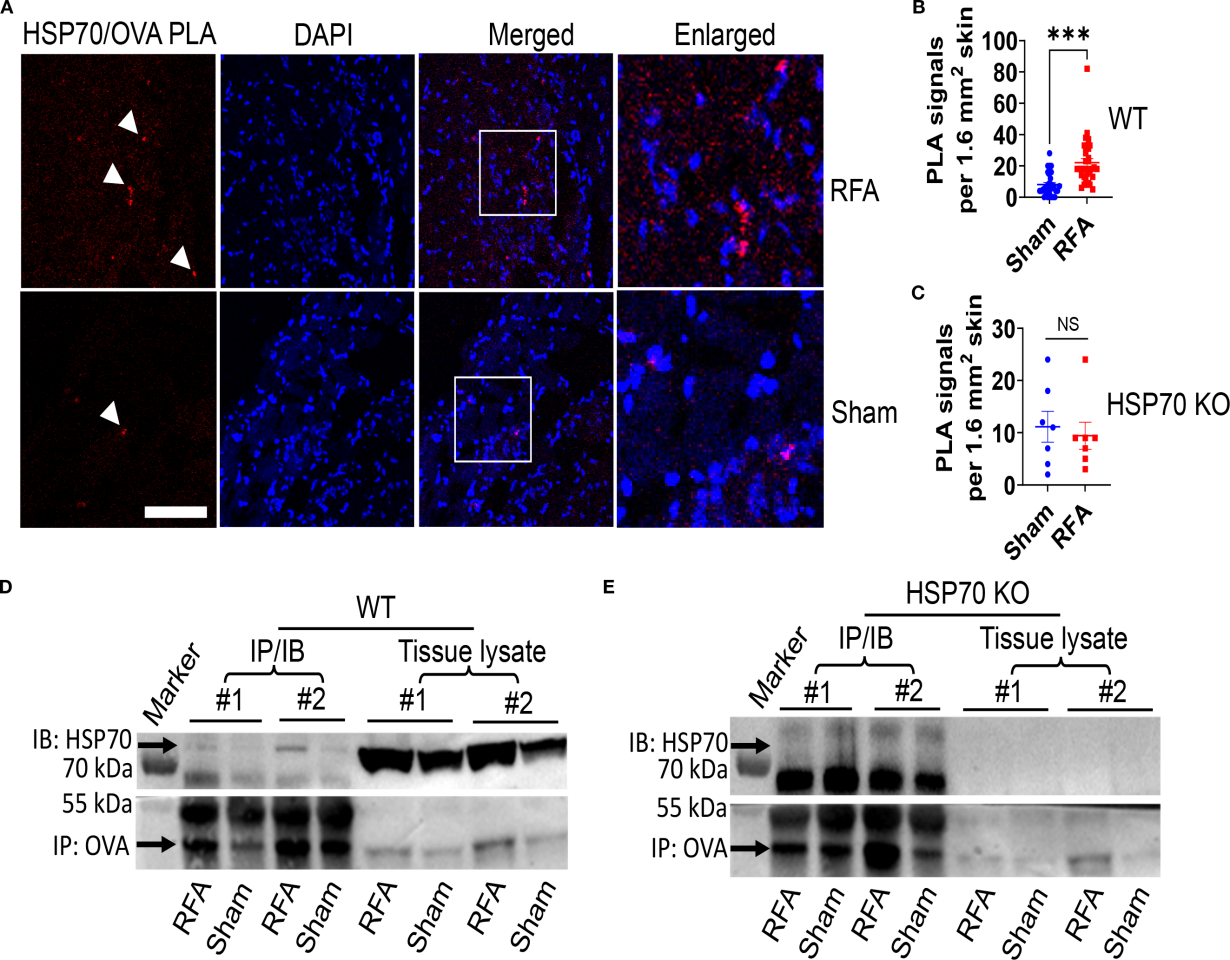
Evidence of *in situ* HSP70/OVA association. Lateral back skin of WT or HSP70 KO mice was subjected to RFA or Sham treatment followed by ID injection of 1 µg OVA into RFA or Sham-treated skin. Skin was collected 6 h later. **(A–C)** Skin was subjected to cryo-sectioning and PLA analysis of HSP70/OVA binding. Representative HSP70/OVA PLA results in WT mice were shown in **A**. Quantitative analysis of HSP70/OVA PLA signals in randomly selected regions of 1.6 mm^2^ in WT mice and HSP70 KO mice was shown in **(B, C)**, respectively. **(D, E)** Lateral back skin of WT and HSP70 KO mice (n=2) were exposed to RFA (left) or Sham treatment (right) followed by ID OVA injection. Skin was collected 6 h later and homogenized in RIPA buffer. The same amounts of total proteins were incubated with anti-OVA antibodies and then protein A/G agarose followed by centrifugation and washing. After boiling, IP samples and tissue lysates were subjected to SDS-PAGE and IB detection of HSP70 and OVA in WT **(D)** and HSP70 KO mice **(E)**. Intact membrane pictures were shown in **Supplementary Figure S4**. Arrows in **A** point to positive PLA signals. Scale in **A**: 100 µm. Two-tailed student’s t-test was used to compare differences between groups in **(B, C)**. n=31–33 in **B** and n=7 in **C**. ***, p<0.001. NS, not significant. Data are representative of two independent experiments with similar results.

Our recent studies found HSP70 was crucial for RFA to enhance ID vaccine-induced immune responses (41, 51). Extracellular release of RFA-induced HSP70 might encounter intradermally injected antigen and facilitate its uptake by APCs. Considering a close association of HSP70 and antigenic peptides or vaccine antigens was necessary to elicit potent adaptive immune responses (3), we attempted to detect association of RFA-induced HSP70 and ID model antigen OVA via a highly sensitive proximity ligation assay (PLA) (52). PLA utilizes DNA probe-conjugated antibodies to study protein/protein interactions. A close proximity of the targets brings together DNA probes to form a circular DNA, which can be amplified by rolling cycle PCR for easy detection by fluorescent probes (52). As shown in **Figure 4A**, more HSP70/OVA PLA signals were found in RFA than Sham-treated skin of WT mice. Quantitative analysis found HSP70/OVA PLA signals were significantly increased after RFA treatment (22 vs. 8, p<0.001) (**Figure 4B**). As expected, HSP70/OVA PLA signals showed no significant difference between RFA and Sham groups in HSP70 KO mice (**Figure 4C**).

Immunoprecipitation (IP) and immunoblotting (IB) were then used to confirm the above findings. As shown in **Figure 4D**, more HSP70 was detected in RFA than Sham-treated skin in both mice (#1 & #2) after IP/IB. More HSP70 in tissue lysates of RFA than Sham-treated skin indicated RFA induced HSP70 synthesis (**Figure 4D**). Interestingly, significantly more OVA was also detected in RFA than Sham-treated skin (**Figure 4D**), hinting RFA might help to retain OVA at local injection sites. Furthermore, the relative HSP70 to OVA levels significantly increased by RFA treatment (**Supplementary Figure S5**), supporting HSP70/OVA binding. We also conducted the same experiments in HSP70 KO mice and failed to detect HSP70 after IP/IB in either RFA or Sham-treated skin (**Figure 4E**). Our results indicate the ability of RFA-induced HSP70 to bind ID OVA *in situ*.

### HSP70 contributes to enhanced antigen uptake in skin and draining lymph nodes

**Figure 5:**
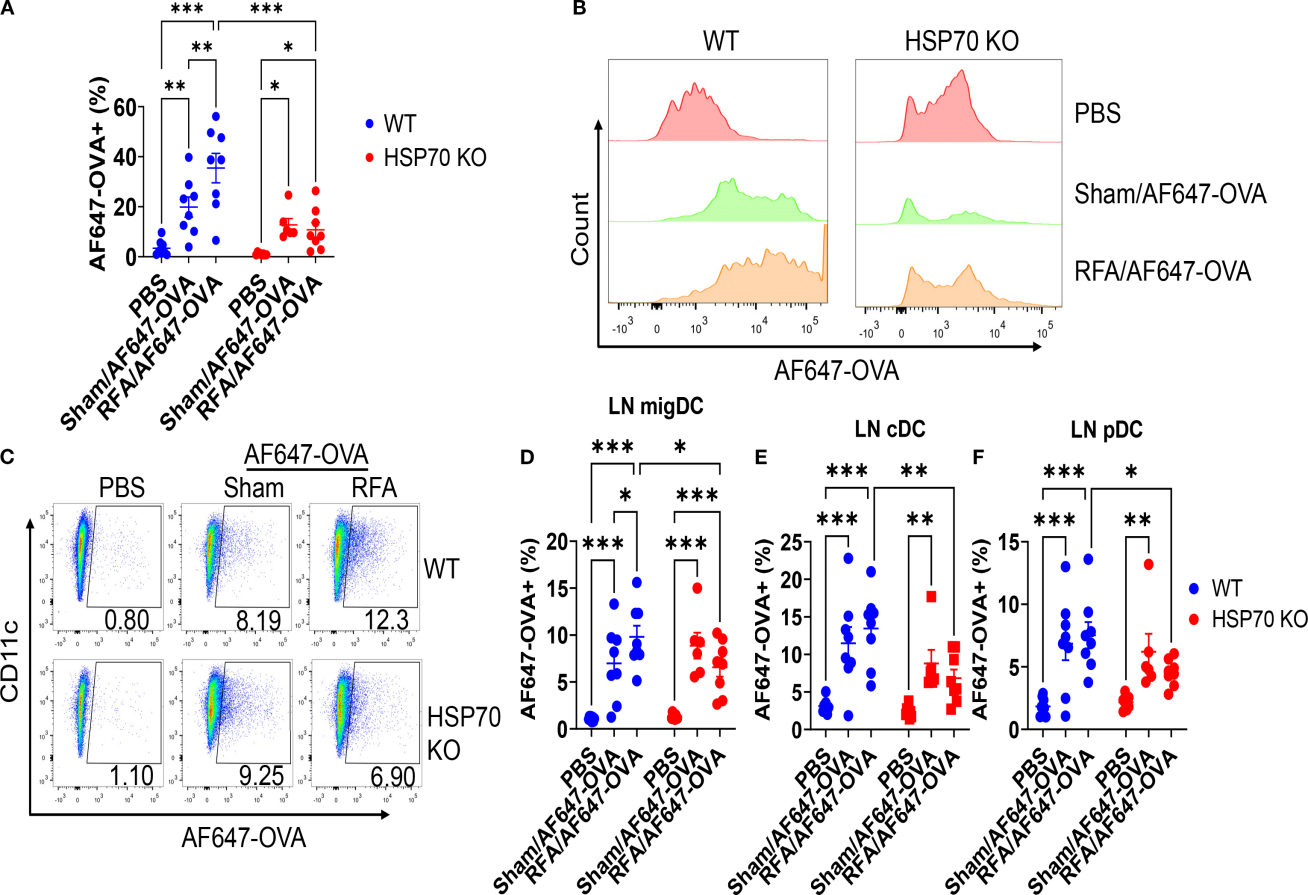
HSP70 contributes to RFA-enhanced antigen uptake in skin and draining LNs. WT and HSP70 KO were subjected to RFA or Sham treatment followed by ID injection of 2 µg AF647-OVA into RF or Sham-treated skin. Skin and draining LNs were dissected 20 h later followed by single-cell suspension preparation, immunostaining, and flow cytometry analysis. Gating strategies were shown in **Supplementary Figures S1B, C**. **(A)** Percentages of AF647-OVA+ cells in skin DCs (CD11c+ MHC II+) in WT and HSP70 KO mice. **(B)** Representative histogram of AF647-OVA levels in skin DCs of different groups in WT and HSP70 KO mice. **(C)** Representative dot plots of AF647-OVA+ migDCs of different groups in WT and HSP70 KO mice. **(D)** Percentage of AF647-OVA+ cells in migDC **(D)**, cDC **(E)**, and pDC **(F)** in WT and HSP70 KO mice. Two-way ANOVA with Fisher’s LSD test was used to compare differences between groups. n=8. *, p<0.05; **, p<0.01; ***, p<0.001. Data are representative of two independent experiments with similar results.

*In situ* HSP70/OVA association hinted RFA-induced HSP70 might facilitate antigen uptake. To corroborate this, WT and HSP70 KO mice were subjected to RFA or Sham treatment followed by ID injection of 2 µg AF647-OVA into RFA or Sham-treated skin. AF647-OVA^+^ DCs were evaluated 18 h later in skin and draining LNs. As shown in **Figure 5A**, RFA significantly increased percentage of AF647-OVA^+^ cells in DCs of WT mice (35 vs. 20%), while failed to increase percentage of AF647-OVA^+^ cells in DCs of HSP70 KO mice (13 vs. 11%). AF647-OVA levels in skin DCs were significantly increased after RFA treatment in WT but not HSP70 KO mice (**Figure 5B**). These results indicated HSP70 played a crucial role in RFA-enhanced antigen uptake in the skin. Skin DC subtype analysis found HSP70 mainly enhanced antigen uptake in dermal DC subsets (I, III, IV) but not epidermal Langerhans cells (DC subset II) (**Supplementary Figure S6**).

Antigen uptake was further analyzed in draining LNs, in which DCs were divided into 3 subtypes (cDC, migDC, and pDC) based on the relative CD11c and MHC II expression as shown in our prior studies (40, 43). As shown in **Figures 5C, D**, RFA significantly increased the percentage of AF647-OVA+ cells in migDC of WT mice (7.0 vs. 9.8%). RFA also increased the percentage of AF647-OVA+ cells in cDC (11.5 vs. 13.4%) and pDC (6.9 vs. 7.5%) of WT mice, but such a difference didn’t reach statistically significant levels (**Figures 5E, F**). Interestingly, RFA slightly reduced percentages of AF647-OVA+ cells in migDC (8.9 vs. 6.6%), cDC (8.8 vs. 6.8%), or pDC (6.2 vs. 4.5%) in HSP70 KO mice (**Figures 5D–F**). These results strongly support crucial roles of HSP70 in RFA-enhanced antigen uptake in draining LNs in particular in migDC.

### HSP70 induces no significant DC maturation *in vivo* or *in vitro*

**Figure 6:**
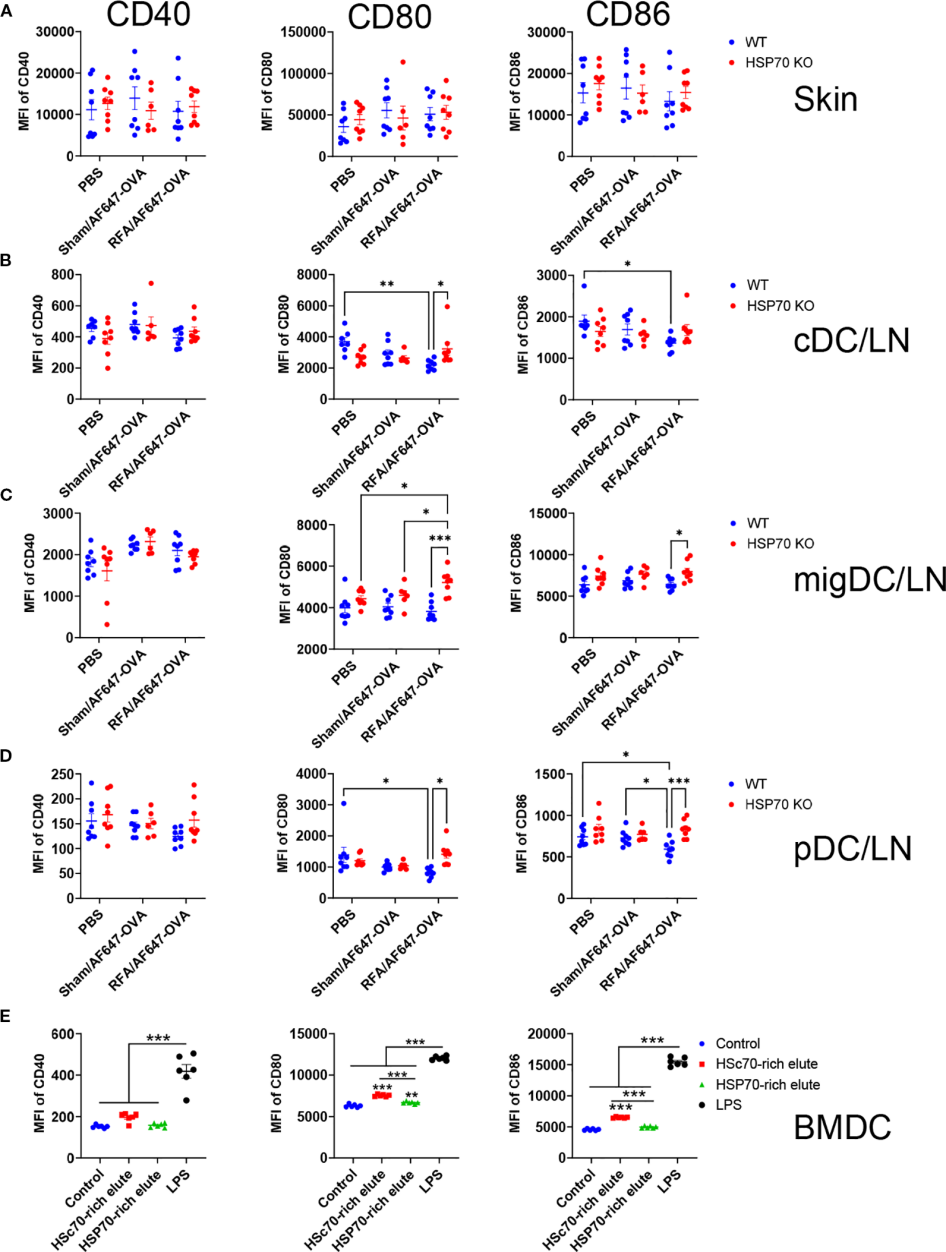
RFA-induced HSP70 lacks the ability to induce DC maturation. **(A)** The same skin cell samples in **Figures 5A, B** were analyzed for expression of surface co-stimulatory molecules (CD40, CD80, and CD86) in CD11c+ DCs of WT and HSP70 KO mice. MFI of CD40, CD80, and CD86 was compared between groups. **(B–D)**. The same LN samples as in **Figures 5C, D** were analyzed for expression of surface co-stimulatory molecules (CD40, CD80, and CD86) in cDC **(B)**, migDC **(C)**, and pDC **(D)** of WT and HSP70 KO mice. MFI of CD40, CD80, and CD86 was compared between groups. **(E)** BMDCs were incubated with AF647-OVA alone (control) or in the presence of HSc70 or HSP70-rich elutes or LPS. Cells were analyzed for their surface expression of CD40, CD80, and CD86 24 h later. MFI of CD40, CD80, and CD86 was compared between groups. Two-way ANOVA with Newman-Keuls’s multiple comparison test was used to compare differences between groups in A-D. One-way ANOVA with Tukey’s multiple comparison test was used to compare differences between groups in **(E)** n=6–8 in A-D. n=6 in **(E)** *, p<0.05; **, p<0.01; ***, p<0.001. Data are representative of two independent experiments with similar results.

The ability of HSP70 to induce DC maturation has been controversial due to the potential contamination of LPS in HSP70 samples. *In vivo* induction of HSP70 by RFA introduces no foreign materials into the body and provides a unique opportunity to study the potential impact of *in situ* generated HSP70 on DC maturation. We analyzed this in the above studies when we evaluated the potential impact of RFA-induced HSP70 on antigen uptake. As shown in **Figure 6A**, RFA failed to increase MFI of CD40, CD80, or CD86 of skin DCs in WT or HSP70 KO mice. In draining LNs, RFA treatment failed to significantly change MFI of CD40 in cDC, migDC, or pDC in WT or HSP70 KO mice (**Figures 6B–D**). Interestingly, lack of HSP70 significantly increased MFI of CD80 in cDC, migDC, and pDC and MFI of CD86 in migDC and pDC in draining LNs of RFA group (**Figures 6B–D**). Furthermore, significantly lower expressions of CD80 and CD86 were observed in cDC and pDC in RFA group when compared to PBS group (**Figures 6B–D**). These results hinted RFA-induced HSP70 might suppress CD80 and CD86 expression in draining LNs of RFA group.

Besides *in vivo* analysis, we also incubated BMDCs with purified HSP70 from RFA (HSP70-rich elutes) or Sham-treated skin (HSc70-rich elutes). LPS was included for comparison. As shown in **Figure 6E**, HSP70-rich elutes slightly induced CD80 expression, while HSc70-rich elutes more potently enhanced CD80 and CD86 expression. As compared to HSc70 or HSP70-rich elutes, LPS more vigorously enhanced CD40, CD80, and CD86 expression on BMDCs. These results indicated the weak ability of HSP70 in stimulation of BMDC maturation in the culture. In this study, we also added AF647-OVA into the BMDC culture to explore whether HSc70 or HSP70-rich elutes could increase antigen uptake by using LPS as the positive control. As shown in **Supplementary Figure S7**, HSc70 or HSP70-rich elutes failed to increase AF647-OVA uptake by BMDCs, while LPS vigorously enhanced AF647-OVA uptake. These results indicated purified HSP70 failed to enhance antigen uptake in BMDC culture. We also explored whether purified HSP70 possessed vaccine adjuvant effects by co-administration with OVA. As shown in **Supplementary Figure S8**, HSc70 or HSP70-rich elutes failed to significantly increase serum anti-OVA antibody titer, while RFA significantly increased serum anti-OVA antibody titer. This result indicated the lack of vaccine adjuvant effects of purified HSP70, potentially due to the lack of antigen binding when simply mixing the two moieties together.

### RFA induces TIRAP/MyD88 association

**Figure 7:**
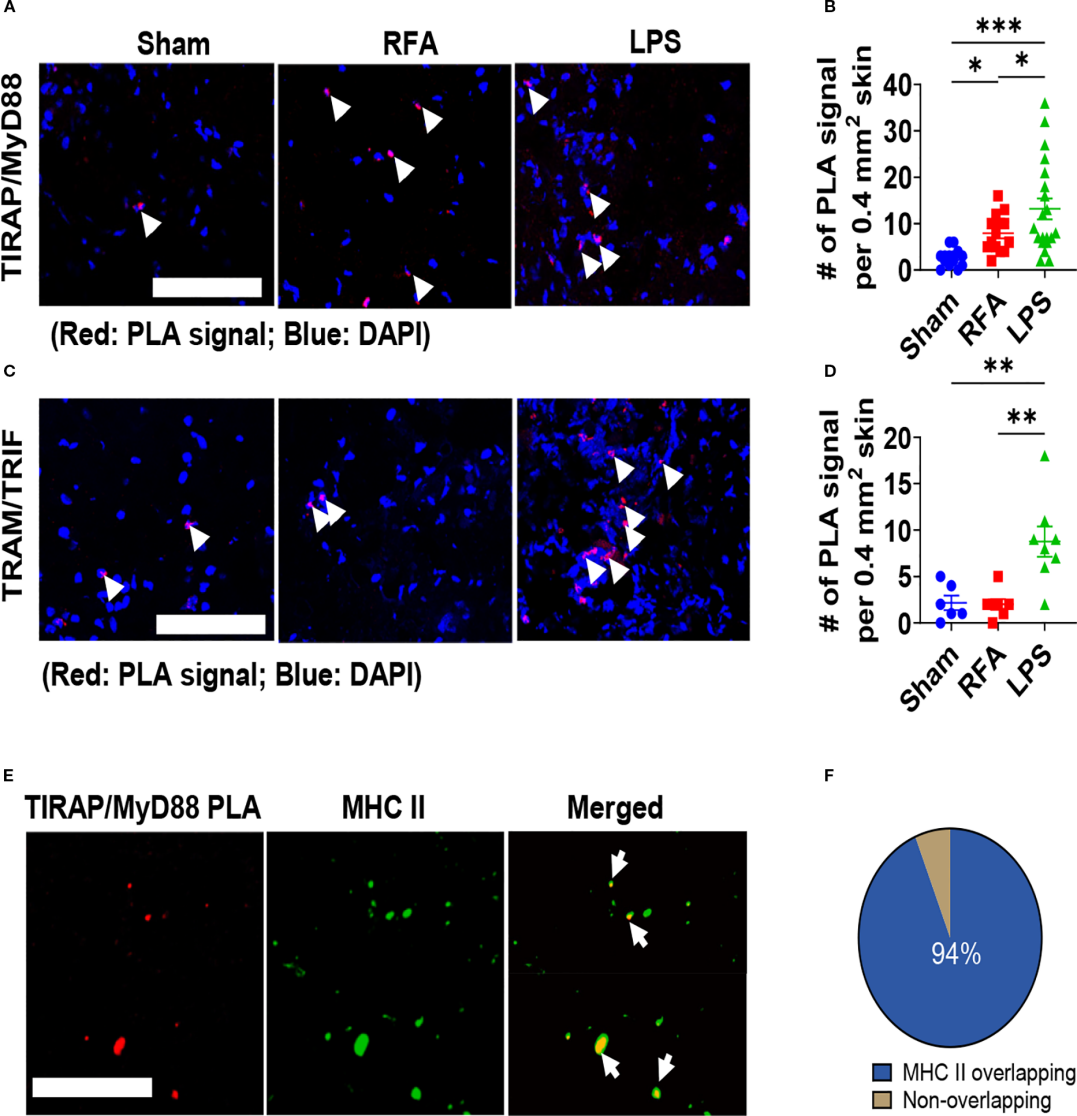
RFA induces TIRAP/MyD88 but not TRAM/TRIF association. **(A–D)**. Mice was subjected to RFA or Sham treatment or ID injection of 5 µg LPS. Skin was collected 6 h later and subjected to cryo-sectioning and PLA analysis of close association of MyD88 and TIRAP **(A, B)** as well as TRIF and TRAM **(C, D)**. Representative PLA images were shown in **(A, C)** and quantitative results were shown in **(B, D)**. Arrows point to PLA signals. **E.** Skin sections in RFA groups in the above studies were also stained with fluorescence-conjugated anti-MHC II antibodies. Z-stack pictures were captured and used to create 3D images. Representative 3D pictures showing the overlapping of RFA-induced TIRAP/MyD88 PLA signals with MHC II. Arrows point to overlapping signals (yellow). **(F)** Pie chart of PLA signals overlapped with MHC II (blue). Scale: 100 µm in **(A, C, E)**. One-way ANOVA with Newman-Keuls multiple comparison test was used to compare differences between groups in **(B, D)**. n=14–20 in **(B)** and n=6–8 in **(D)**. Over 50 PLA signals were explored by investigators in **(F)**. *, p<0.05; **, p<0.01; ***, p<0.001. Data are representative of three independent experiments with similar results.

Our previous studies found MyD88 played a crucial role in RFA effects (40, 41). MyD88 is an essential adaptor downstream of most TLRs except TLR3 (53). Most TLRs directly recruit MyD88, while TLR2 and TLR4 first recruit TIRAP and then MyD88 (53). TLR3 and TLR4 first recruit TRAM and then TRIF (53). To explore whether other adaptors were activated by RFA, we detected TIRAP/MyD88 or TRAM/TRIF association following RFA, Sham, and LPS treatments via tissue PLA (52). As shown in **Figures 7A, B**, RFA significantly increased and LPS more significantly increased TIRAP/MyD88 PLA signals. Interestingly, RFA didn’t increase TRAM/TRIF PLA signals, while LPS significantly increased TRAM/TRIF PLA signals (**Figures 7C, D**). These results indicated RFA induced TIRAP/MyD88 but not TRAM/TRIF association.

Next, we used FITC-conjugated anti-MHC II antibodies to stain DCs and macrophages to explore the relative localization of TIRAP/MyD88 PLA signals. As shown in **Figure 7E**, we captured 3D pictures via Z-Stack and found the majority of RFA-induced PLA signals overlapped with MHC II (**Figure 7E**). Overall, more than 90% PLA signals overlapped with MHC II (**Figure 7F**). This result indicated the majority of RFA-induced TIRAP/MyD88 association occurred in DCs and macrophages, in line with the function of these cells in innate and adaptive immunity.

### HSP70 suppresses RFA-induced TLR4/IRAK/NFκB signaling

**Figure 8:**
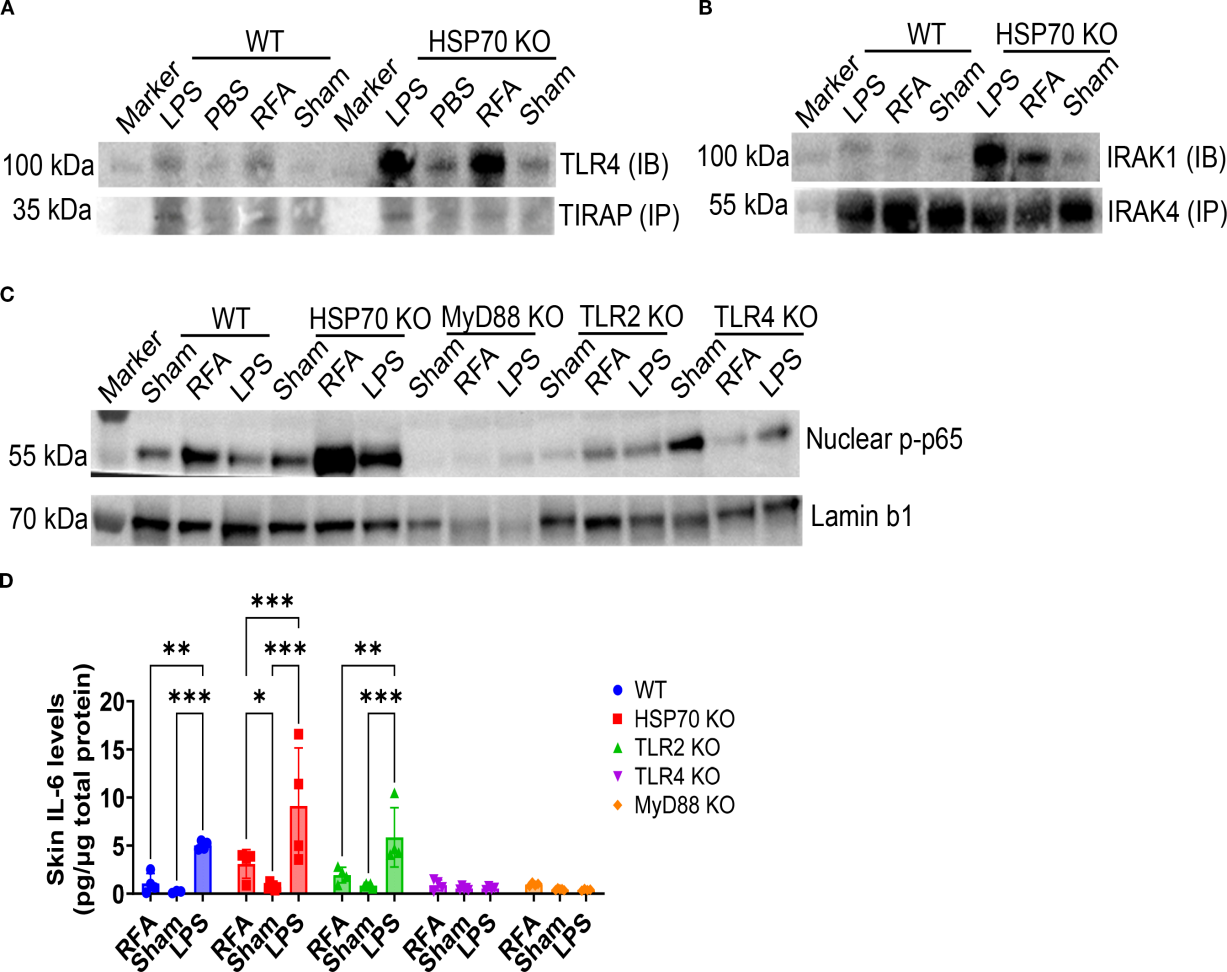
HSP70 suppresses RFA-induced TLR4/IRAK/NFκB signaling. **(A, B)** WT and HSP70 KO mice were subjected to RFA or Sham treatment or ID injection of LPS or PBS. Skin was collected 6 h later in **(A, B)** IP and IB were conducted to evaluate TLR4/TIRAP binding **(A)** and IRAK4/IRAK1 binding in **(B, C)**. WT, HSP70 KO, MyD88 KO, TLR2 KO, and TLR4 KO mice were subjected to RFA or Sham treatment or ID injection of LPS. Skin was collected 2 h later. Cytosol and nuclear fractions were separated and nuclear fraction was analyzed by WB analysis to detect phosphorylated p65 using Lamin b1 as a loading control. **(D)** Skin IL-6 levels 6 h after RFA, Sham, or LPS treatment of lateral back skin of WT, HSP70 KO, TLR2 KO, TLR4 KO, and MyD88 KO mice. Two-way ANOVA with Fisher’s LSD test was used to compare differences between groups. n=4-6. *, p<0.05; **, p<0.01; ***, p<0.001. Original membrane pictures were shown in **Supplementary Figure S9**. Data are representative of two independent experiments with similar results.

The involvement of TIRAP hinted its unique upstream TLR (TLR2 or TLR4) might be activated by RFA. The potential association of TLR4/TIRAP or TLR2/TIRAP was then explored via IP (anti-TIRAP)/IB (anti-TLR2 or TLR4). The underlying roles of HSP70 in potential RFA-induced TLR2 and TLR4 activation were also explored. To this end, WT and HSP70 KO mice were treated with RFA or Sham or intradermally injected with LPS or PBS. As shown in **Figure 8A**, RFA and LPS weakly induced TLR4/TIRAP association in WT mice, while vigorously induced TLR4/TIRAP association in HSP70 KO mice. This result hinted a strong suppression of TLR4/TIRAP association by HSP70. Interestingly, we failed to detect TLR2/TIRAP association with the same method (data not shown), hinting RFA might mainly induce TLR4/TIRAP/MyD88 pathway. TLR4/MyD88 activation has been reported to recruit IRAK4, which auto-phosphorylates and then recruit IRAK1 (54). Subsequently, sequential phosphorylation of Thr209 and Thr387 of IRAK1 leads to hyperphosphorylation of the proline-, serine-, and threonine-rich ProST region followed by dissociation from Myddosome and association with TRAF6 (55). To this end, we detected possible association of IRAK4 and IRAK1 via IP (anti-IRAK4) and IB (anti-IRAK1) in WT and HSP70 KO mice. As shown in **Figure 8B**, RFA and LPS weakly induced IRAK4/IRAK1 association in WT mice, while vigorously induced IRAK4/IRAK1 association in HSP70 KO mice. This result indicated strong suppression of IRAK4/IRAK1 association by HSP70. In our study, we found IRAK1 showed a higher than normal molecular weight (>100 kDa), hinting a hyperphosphorylation status. We also directly detected phosphorylated p65 in nucleus, the key event of NFκB activation downstream of TLR4/MyD88 activation. We included various KO mice to explore whether HSP70, TLR2, TLR4, MyD88 played a role in this process. As shown in **Figure 8C**, RFA induced nuclear transmigration of phosphorylated p65 in WT mice. This process was profoundly enhanced in HSP70 KO mice, hinting HSP70 strongly suppresses this process. Lack of TLR4 and MyD88 but not TLR2 abrogated this process, hinting the crucial roles of TLR4 and MyD88 but not TLR2 in activation of phosphorylated p65 nuclear transmigration. Our previous studies found RFA could induce temporary IL-6 gene expression (40). Considering IL-6 gene expression is controlled by NFκB (56), we also measured skin IL-6 levels 6 h after RFA, Sham, or LPS treatment. As shown in **Figure 8D**, RFA induced low levels of IL-6 expression in WT mice (1.07±0.52 pg/µg) and about 3-fold higher IL-6 expression in HSP70 KO mice (3.10±0.75 pg/µg). Skin IL-6 levels were significantly higher in RFA than Sham group in HSP70 KO mice but not WT mice, indicative of a suppressive role of HSP70 in IL-6 expression. Similar to WT mice, RFA induced weak IL-6 expression in TLR2 KO, TLR4 KO, and MyD88 KO mice (**Figure 8D**). As expected, LPS more significantly increased skin IL-6 levels than RFA in WT, HSP70 KO, and TLR2 KO mice and failed to increase skin IL-6 levels in TLR4 KO and MyD88 KO mice considering LPS stimulates TLR4/MyD88-mediated NFκB activation (57). Significantly increased skin IL-6 levels after RFA or LPS treatment in HSP70 KO mice as compared to WT mice hinted HSP70-mediated suppression was shared between RFA and LPS activators. Taken together, our data indicated RFA-induced TLR4/MyD88/NFκB pathway was strongly inhibited by inducible HSP70.

## Discussion

Our study revealed dual roles of RFA-induced HSP70 in antigen delivery and immunomodulation. RFA-induced HSP70 promoted ID antigen uptake by skin and LN DCs, while at the same time suppressed RFA-induced NFκB activation and IL-6 cytokine release. Our study identified the roles of RFA-induced HSP70 in RFA effects and also explained RFA-induced transient and low-level local inflammation (40). These findings add to our increased understanding about the functional roles of inducible HSP70 *in vivo*.

RFA-induced HSP70 formed complexes with intradermally injected OVA. HSP70/OVA complexes were stable and endured skin homogenization and immunoprecipitation processes. This finding is surprising considering the majority of the prior studies found simply mixing HSP70 and peptides could not form complexes (3, 58). HSP70/peptide complex formation requires an appropriate buffer and following specific incubation steps (6, 58). Furthermore, HSP70 needs to be stripped of associated peptides prior to accept new peptides (3). It seems RFA treatment creates a suitable environment to facilitate HSP70/OVA complex formation. It remains to be explored whether RFA is unique or *in situ* induced HSP70 in general has the ability to bind vaccine antigens. We believe RFA-induced HSP70 can also bind other vaccine antigens, such as influenza vaccine antigens (e.g., hemagglutinin antigen), considering their immunogenicity was significantly increased by RFA (40–42). More studies are needed to identify key regions of HSP70 responsible for antigen binding and antigen regions responsible for HSP70 binding. HSP70/OVA binding most likely occurs in extracellular space, which is supported by our observation that RFA-induced HSP70 could be released into extracellular space. Extracellular HSP70/OVA binding is expected to facilitate antigen transportation to MHC I and II presentation pathways and elicit potent humoral and cellular immune responses as observed in our previous studies (40). RFA-induced HSP70/OVA binding also explained the significant dose-sparing of RFA considering HSP70 bound peptide only requires sub-nanograms to elicit potent immune responses (3, 41). This finding support induction of *in situ* HSP70 synthesis to enhance vaccine-induced immune responses without the need to prepare HSP70/antigen complexes with endotoxin contamination risks.

RFA-induced HSP70 played a major role in immunosuppression rather than immunostimulation. Lack of HSP70 significantly increased TLR4/TIRAP and IRAK4/IRAK1 binding, nuclear translocation of phosphorylated p65, and IL-6 expression after RFA treatment. We believe an endogenous TLR4 agonist might be triggered by RFA that contributed to TLR4/IRAK/NFκB activation (59). The endogenous TLR4 agonist should only stimulate TIRAP/MyD88 but not TRAM/TRIF activation. Surprisingly, HSP70 also played an inhibitory role in LPS-induced TLR4/IRAK/NFκB activation. A literature search found LPS could induce significant HSP70 synthesis in monocytes or macrophages (60, 61). Prior studies found HSP70 played critical roles in inhibition of IκBα degradation and nuclear translocation of p65 (28), proteasome degradation of p65 in cell nucleus (62), TRAF6 binding and ubiquitination prevention (26), providing possible mechanisms to the observed inhibitory roles of HSP70 in our studies. Our prior studies found RFA only induced transient, low-level local inflammation without provoking strong local inflammation as observed with chemical adjuvants (40). We believe the good local safety is at least partly due to the immunosuppressive roles of RFA-induced HSP70. RFA-induced HSP70 induced little or no DC maturation and instead slightly suppressed DC maturation *in vivo*. In support, lack of HSP70 was associated with significantly increased CD80 and CD86 expression in DC subsets in draining LNs following RFA treatment. Purified HSP70 slightly enhanced CD80 expression of BMDCs *in vitro* with the potency much weaker than LPS. Our study support the major role of HSP70 in immunoregulation rather than immunostimulation.

Our previous studies found MyD88 played crucial roles in RFA effects. This study found RFA could activate TIRAP/MyD88 association, which can lead to NFκB activation and DC maturation. Yet, no significant DC maturation was observed in our studies. Our unpublished data indicated MyD88 was not critical to RFA-enhanced antigen uptake. We believe MyD88 acted through yet unidentified mechanisms to enhance RFA-induced immune responses. Interestingly, MyD88 was found to be crucial for MF59 adjuvant to enhance vaccine-induced antibody responses although MF59 was known not to activate TLRs (63). In our previous studies, we found MyD88 was critical for ablative fractional laser (AFL) adjuvant effects to enhance ID vaccine-induced antibody responses (43). These two studies hint the existence of yet unknown mechanisms of MyD88 activation to enhance vaccine-induced immune responses.

Our study found skin DCs were highly sensitive to RFA treatment to increase HSP70 levels, which might be critical for the observed RFA effects considering vaccines can be quickly drained away from the injection site. The underlying mechanism of the rapid increase of HSP70 levels in DCs remains to be explored and may be due to preexisting HSP70 mRNA in skin DCs. In support, prior studies found appreciable levels of HSP70 mRNA present in monocytes and granulocytes but not lymphocytes at the baseline (60). Besides a rapid increase of HSP70 levels, DCs showed the highest HSP70 synthesis as compared to macrophages and non-immune cells. It enables a sufficient amount of HSP70 to accumulate in extracellular milieu of DCs for efficient antigen capturing and intracellular transportation.

In summary, this study discovered dual roles of *in situ* generated HSP70 in antigen delivery and immunomodulation. Our findings can be used to explain the systemic anti-tumor immunity observed after radiofrequency tumor ablation, which stimulates HSP70 release from sublethal thermal damage regions to potentially uptake released tumor antigens from dying cells and presentation to MHC I pathway to stimulate anti-tumor immunity (64). Our findings suggest that endogenously induced HSP70 can serve as an *in situ* chaperone for encountering antigens, offering an explanation on the potent dose-sparing effects of RFA, which enabled nanograms of influenza vaccines to elicit protective immunity (41). Our findings supports the induction of *in situ* HSP70 to potentiate ID vaccine-induced humoral and cellular immune responses to prevent infectious diseases or in tumor therapy. On the other end, targeted induction of HSP70 may offer therapeutic potential in autoimmune diseases by dampening NFκB-driven cytokine release. These dual functions highlight opportunities to exploit endogenous HSP70 for both vaccine adjuvantation and immunomodulation.

## Data Availability

The raw data supporting the conclusions of this article will be made available by the authors, without undue reservation.
