# Supplementary material for: Dual roles of in situ generated HSP70 in antigen delivery and immunoregulation
Source: Front Immunol. 2025 Oct 2;16:1638948. doi: 10.3389/fimmu.2025.1638948 (PMC12528137; doi:10.3389/fimmu.2025.1638948)
Supplement: Supplementary file 1 [file SupplementaryFile1.docx]

**Supplementary Information**

**Dual roles of in situ generated HSP70 in antigen delivery and immunoregulation**

**Kang et al.**

**
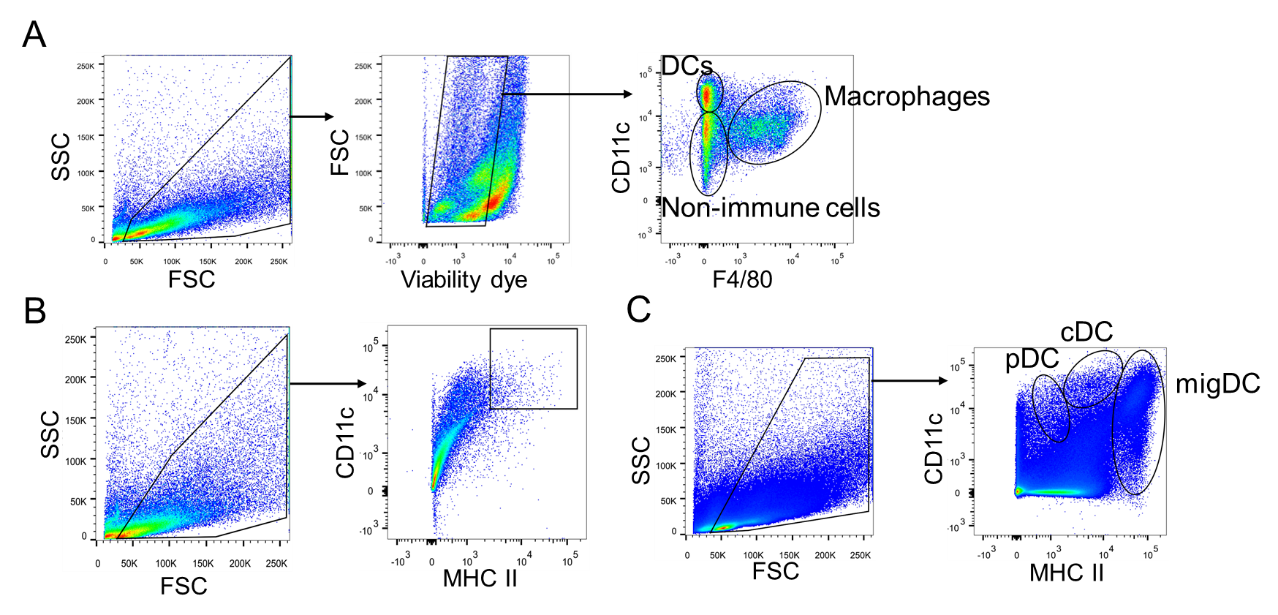
**

**Fig.S1 Flow cytometry gating strategies**

A. Gating strategies for Fig.1. Live cells were gated based on SSC and FSC. Cells were then gated to exclude viability dye^+^ dead cells. Cells were lastly gated based on CD11c and F4/80 expression. B. Gating strategies for Fig.5B. Live cells were gated based on SSC and FSC. Cells were then gated based on CD11c and MHC II expression. Cells were than analyzed for HSP70 expression. C. Gating strategies for Fig.5C. Live cells were gated based on SSC and FSC. Cells were then gated based on CD11c and MHC II expression to differentiate migDC, cDC, and pDC.


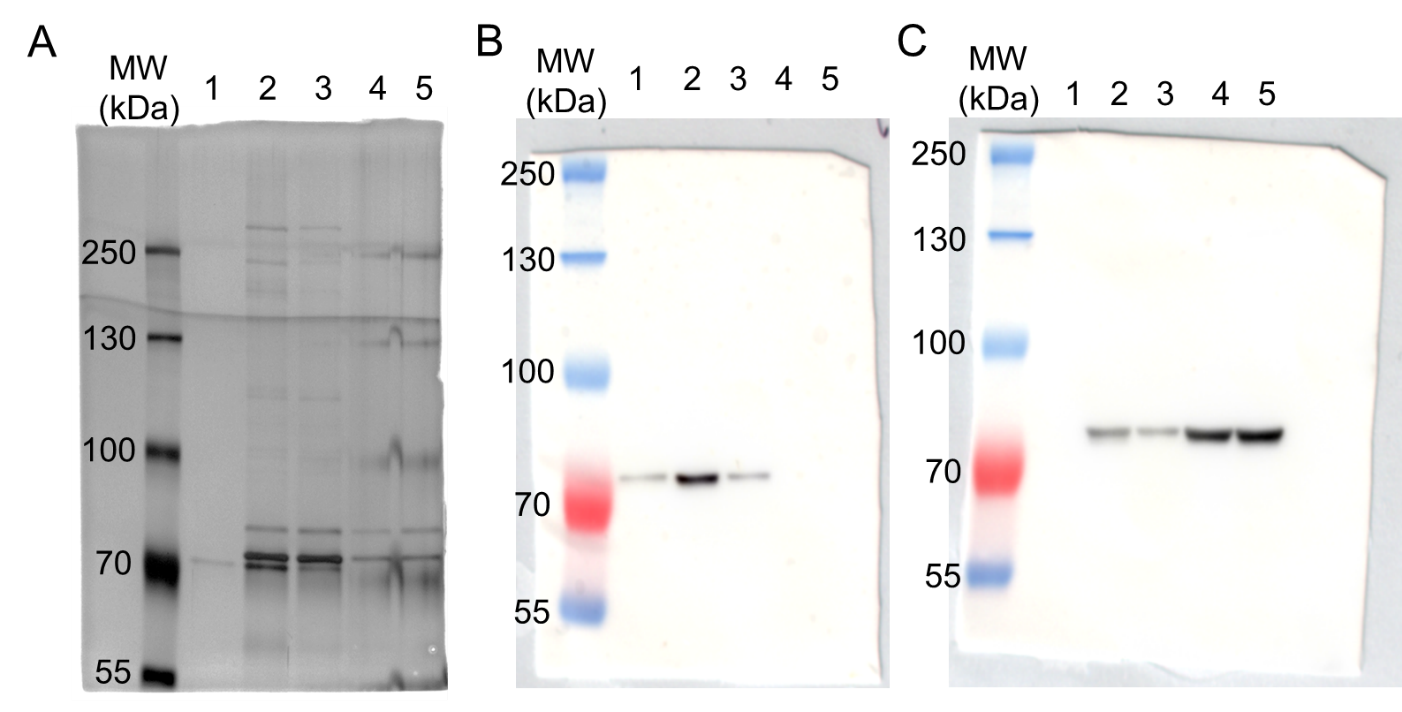


**Fig.S2 Whole membrane pictures of Fig.2A (A), Fig.2B (top, B), and Fig.2B (bottom, C)**

**Fig.S3 Membrane HSP70+ cells following RFA or Sham treatment**

Lateral back skin of C57BL/6 mice was subjected to RFA or Sham treatment. RFA and Sham-treated skin was dissected 24 h later followed by single-cell suspension preparation, immunostaining with cmHSP70 antibodies (specifically recognizing membrane bound HSP70) or isotype control, and flow cytometry analysis. n=4

**
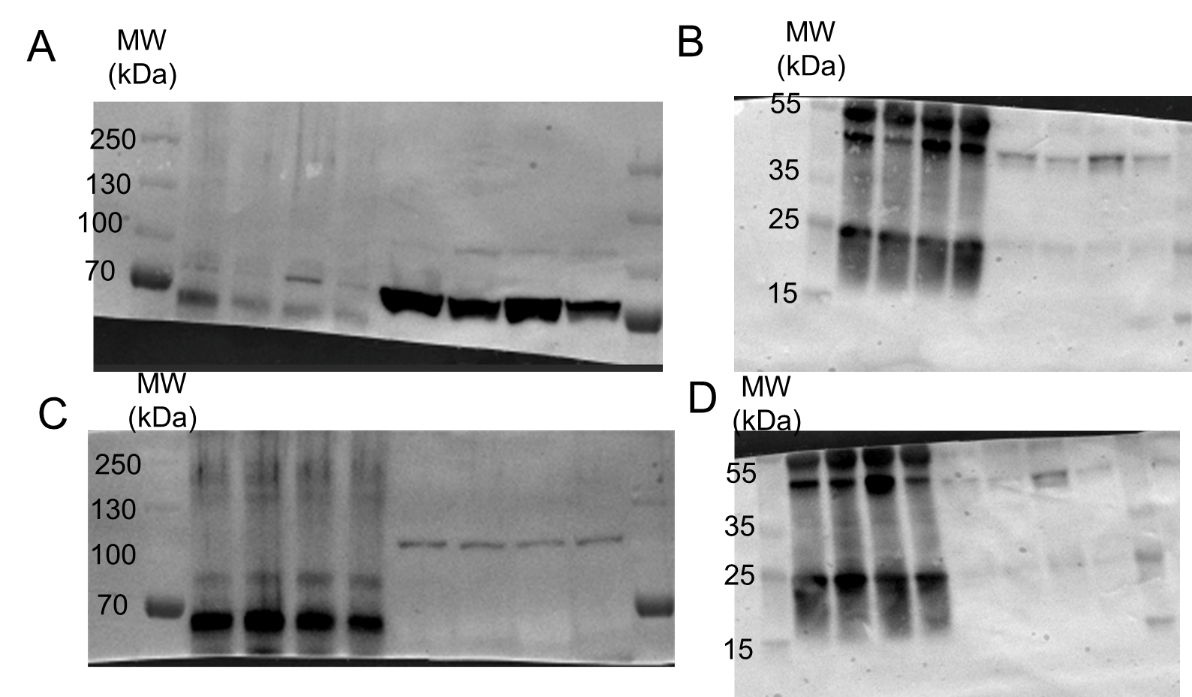
**

**Fig.S4 Intact membrane pictures of Fig.4D (A, B) and Fig.4E (C, D)**

**Fig.S5 Relative HSP70 to OVA levels**

Densitometry analysis of the relative HSP70 to OVA levels in Fig.4D. Results were the combination of two independent experiments with similar results. Two-tailed student’s t-test was used to compare differences between groups. *, p<0.05.


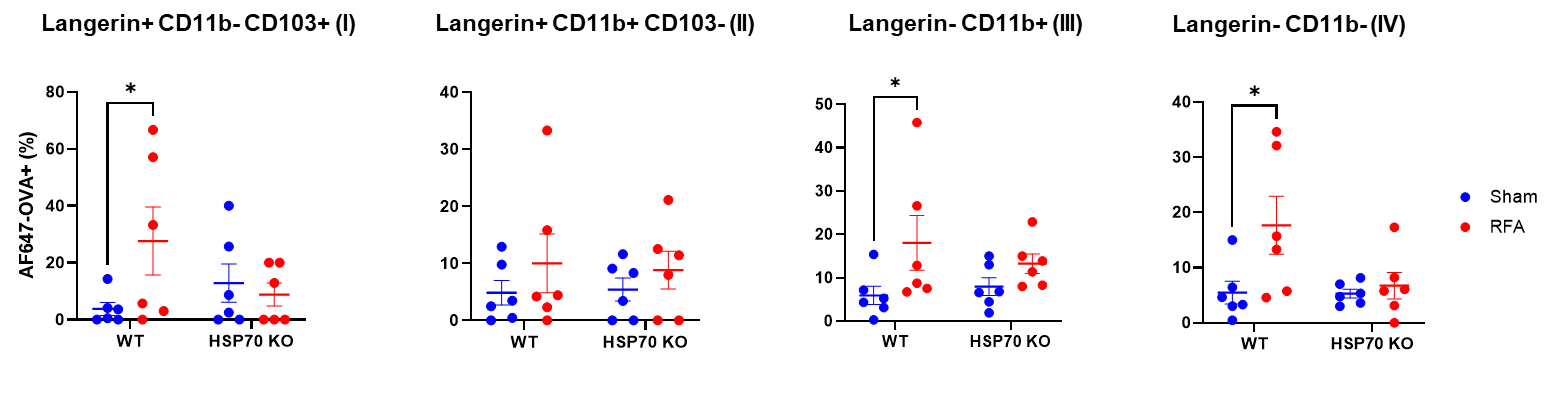


**Fig.S6 HSP70 is crucial for RFA-enhanced antigen uptake in dermal DC subsets**

Lateral back skin of WT and HSP70 KO mice was subjected to RFA or Sham treatment followed by ID injection of 2 µg AF647-OVA into RFA or Sham-treated skin. RFA and Sham-treated skin was dissected 18 h later followed by single-cell suspension preparation, immunostaining, and flow cytometry analysis. Skin DCs were divided into 4 groups based on relative expression of Langerin, CD11b, and CD103: Langerin^+^ CD11b^-^ CD103^+^ (I), Langerin^+^ CD11b^+^ CD103^-^ (II), Langerin^-^ CD11b^+^ (III), and Langerin^-^ CD11b^-^ (IV). Percentage of AF647-OVA^+^ cells in DC subsets was compared between RFA and Sham groups in WT and HSP70 KO mice. Two-way ANOVA with Fischer’s LSD test was used to compare differences between groups. n=6. *, p<0.05.

**Fig.S7 HSP70 slightly increased antigen uptake in BMDCs**

Lateral back skin of C57BL/6 mice was subjected to RFA or Sham treatment. RFA and Sham-treated skin was harvested 18 h later and subjected to ATP-Agarose affinity purification to obtain HSP70 or HSc70-rich elute. BMDCs were then incubated with AF647-OVA in the presence or absence of HSP70 or HSc70-rich elute or medium control or LPS. BMDCs were harvested 24 h later and subjected to immune-staining and flow cytometry analysis of percentages of AF647^+^ cells in CD11c^+^ DCs. n=6. One-way ANOVA with Tukey’s multiple comparison test was used to compare differences between groups. *, p< 0.05; ***, p<0.001.

**Fig.S8 Lack of adjuvant effects of purified HSP70**

C57BL/6 mice were subjected to RFA or Sham treatment. RFA and Sham-treated skin was harvested 18 h later. Skin was homogenized and then subjected to ATP-Agarose column purification. HSP70-rich elute was concentrated to 100 µl. C57BL/6 mice were subjected to RFA or Sham treatment followed by ID injection of 10 µg OVA alone (no adjuvant), or in the presence of 25 µl purified HSP70-rich elute from RFA-treated skin or 25 µl purified HSc70-rich elute from Sham-treated skin or with prior RFA treatment. Serum anti-OVA antibody titer was measured 3 weeks later. n=4. One-way ANOVA with Kruskal-Wallis multiple comparison test was used to compare differences between groups. *, P < 0.05.


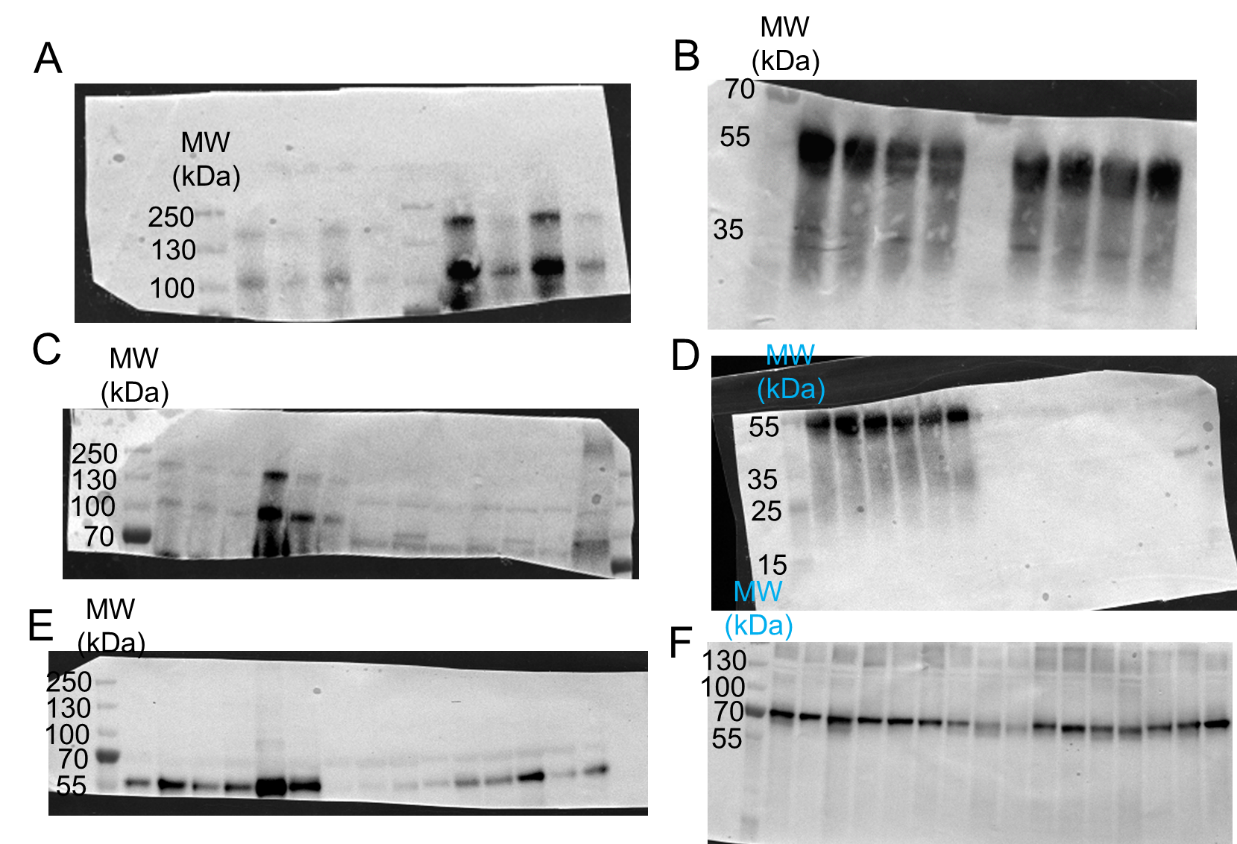


**Fig.S9 Original membrane pictures of Fig.8A (A, B), Fig.8B (C, D), and Fig.8C (E, F)**
